# High neutrophil-lymphocyte ratio indicates a worse response to ursodeoxycholic acid in primary biliary cholangitis: a retrospective cohort study

**DOI:** 10.1186/s12876-023-03031-8

**Published:** 2023-11-17

**Authors:** Huiling Zhu, Mengyao Zheng, Haiyu He, Hongtao Lei, Wenlin Tai, Jinhui Yang

**Affiliations:** 1 Department of Gastroenterology, Second Affiliated Hospital of Kunming Medical University, Kunming, China; 2 School of Public Health Kunming Medical University, Kunming, China; 3 Clinical Lab, Second Affiliated Hospital of Kunming Medical University, Kunming, China

**Keywords:** Primary biliary cholangitis, Neutrophil-to-lymphocyte, Prognosis

## Abstract

**Background:**

Primary biliary cholangitis (PBC) is a chronic cholestatic liver disease characterized by inflammation of the interlobular bile ducts. Ursodeoxycholic acid (UDCA) is the only FDA approved first-line therapy for PBC, but up to 40% of patients with PBC have an incomplete response to UDCA. Neutrophil-to-lymphocyte (NLR) has been used to predict prognosis in various liver diseases. There is limited evidence on the treatment response to UDCA in PBC patients. Our study aimed to evaluate the relationship between NRL and the response to UDCA treatment in PBC patients.

**Methods:**

A total of 257 primary biliary cholangitis (PBC) patients treated with UDCA (13–15 mg/kg/d) were enrolled in this retrospective study. The response to treatment was evaluated based on alkaline phosphatase levels ≤1.67 times the upper limit of the normal value after 12 months of UDCA treatment. Multivariable logistic regression analysis was performed to investigate the association between NLR at baseline and the response to 12 months of UDCA treatment after adjusting for important confounding variables. The stability of the results was evaluated by unadjusted and adjusted models.

**Results:**

The results of multiple regression analysis showed that NLR at baseline was positively associated with the nonresponse to UDCA treatment after adjustments for potential confounders (age, sex, BMI, hypertension, arterial plaque, thyroid disease, jaundice, albumin, globulin, total bile acid, ALP, GGT, LDLC, total cholesterol, hemoglobin, and APTT) (OR = 1.370, 95% CI 1.066–1.761). These results reveal that NLR is an independent risk factor for UDCA treatment nonresponse.

**Conclusions:**

Our results suggest that PBC patients with a high NLR had a worse response to UDCA therapy.

## Introduction

Primary biliary cholangitis (PBC, formerly known as primary biliary cirrhosis) is a chronic and cholestatic autoimmune liver disease characterized by the destruction of intrahepatic small bile ducts, leading to inflammation, fibrosis, hepatic failure, and, in some cases, hepatocellular carcinoma [1, 2] The serologic hallmark of PBC is the anti-mitochondrial antibody (AMA), especially the AMA-M2 subtype, which is a highly disease-specific autoantibody in PBC patients [3]. The presence of this antoantibody has also been shown to precede biochemically apparent liver injury by several years [4]. Antinuclear autoantibodies might be positive in patients with PBC, more specifically antiglycoprotein 210 (anti-gp210) and anti-sp100 [5]. Interestingly, its worldwide prevalence has recently been increasing, and the prevalence has risen to 19.1 cases per 100,000 in Chinese individuals [6, 7]. The first-line therapy in PBC is ursodeoxycholic acid (UDCA) at a dose of 13–15 mg/kg/day, which can significantly improve liver biochemistry, delay histological progression, and improve liver-transplantation-free survival [8]. However, approximately 40% of patients have an inadequate response to UDCA treatment [9]. Compared to responders to UDCA, these patients are at a greater risk of hepatic complications such as ascites, variceal bleeding, and hepatic encephalopathy [10]. At present, there are no reliable clinical approaches to identify UDCA-non-responsive patients before treatment. Therefore, identifying optional biomarkers that can predict either responders or nonresponders to UDCA in early stage have been of tremendous importance to improve clinical decision making. The neutrophil to lymphocyte ratio(NLR) is an easily obtained serum measure that corresponds to systemic inflammation and has been demonstrated to be a valuable prognostic indicator in different types of malignancies [11, 12], inflammatory disease [13], and chronic liver diseases [14–16], However, it is not yet clear whether NRL is associated with poor therapeutic response in PBC patients. Our study aimed to evaluate the relationship between NRL and the response to UDCA treatment in PBC patients.

## Patients and methods

### Study patients

**Fig. 1:**
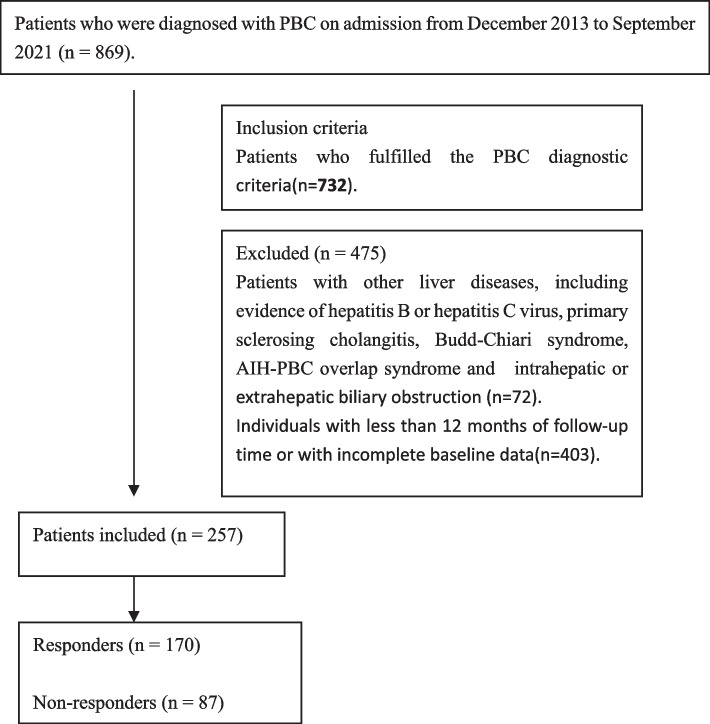
Flowchart of participant selection

This single-center, retrospective cohort study was conducted at the Department of Gastroenterology, Second Affiliated Hospital of Kunming Medical University (Kunming, China). All patients (*n* = 732) were diagnosed with PBC on admission from December 2013 to September 2021, and their data were collected via the electronic medical record database. Patients who fulfilled the inclusion criteria were enrolled, and their demographics and clinical data were extracted from the individual medical records in a case report form. Summary statistical analyses were employed to examine the baseline demographics at their first diagnosis of PBC (Fig. 1).

The present study protocol was implemented in accordance with the ethical guidelines of the Declaration of Helsinki and was approved by the Ethics Committee of the Second Affiliated Hospital of Kunming Medical University. Because we collected already existing ordinary clinical data, ethical clearance of this retrospective study was obtained and the requirement for informed consent was waived (YJ-2023-96).

### Diagnostic criteria

According to the Guideline of the American/European Association for the Study of Liver Disease, patients could be diagnosed with PBC when two of the following three criteria are fulfilled: (1) elevated ALP in the liver biochemical indicators; (2) positive serum AMA (especially the AMA-M2 subtype) or specific antinuclear antibody (sp100, gp210); and (3) manifestations of nonsuppurative cholangitis and intrahepatic interlobular bile duct destruction established through liver biopsy [17, 18].

### Inclusion criteria

The following inclusion criteria were applied: (1) patients who fulfilled the PBC diagnostic criteria; (2) patients with or without previous oral UDCA administration history, who began to take 13–15 mg/kg/d UDCA orally from the baseline date and received continuous and regular treatment; and (3) qualified patients with complete data at baseline and at least a 12-month follow-up period duration.

### Exclusion criteria

Patients who fulfilled any one of the following criteria were excluded: evidence of hepatitis B or hepatitis C virus, primary sclerosing cholangitis or other liver disease, including Budd-Chiari syndrome, autoimmune hepatitis-primary biliary cholangitis (AIH-PBC) overlap syndrome, and intrahepatic or extrahepatic biliary obstruction, and patients who were followed up for less than 1 year. Initially, patients who fulfilled any of the exclusion criteria were also excluded from this study (*n* = 475). Finally, we retrospectively included 257 patients who met the inclusion criteria.

### Data collection

All clinical and laboratory data were recorded upon patient enrollment, including demographic characteristics, basic disease, clinical symptoms, clinical data, and laboratory test data. Specifically, the course of disease, history of UDCA treatment, cirrhotic status, and comorbidities were recorded. Patients who had not used UDCA before and began to use it at baseline were defined as first-time users of UDCA (F-UDCA). The endpoint was UDCA response, defined as alkaline phosphatase < 1.67 times the upper limit of the normal value after 12 months of UDCA treatment. The NLR was measured by dividing the absolute neutrophil count by the lymphocyte count.

Because the data contains patient information. The datasets used and/or analysed during the current study available from the corresponding author on reasonable request.

### Statistical analysis

All analyses adopted a significance level of 0.05 and were conducted in SPSS 26.0 software. Data with a normal distribution were expressed as mean ± SD, whereas those not conforming to a normal distribution were expressed as the median (IQR). Frequencies and percentages were used to express categorical qualitative variables. Data with a normal distribution with homogeneity of variance were compared using an independent sample t-test. Mann-Whitney’s nonparametric test was employed to compare nonnormally distributed between groups. Nonparametric X^2^-tests or Fisher’s exact tests were implemented to compare categorical variables.

Variables that were identified as statistically significant by univariate analyses (*P* < 0.05) or clinically significant were entered into a multivariate logistic regression analysis to determine the contribution of NLR to the endpoint of UDCA response. Multiple logistic regression analysis was utilized to assess the specific relations between the neutrophil-to-lymphocyte ratio (NLR) and outcome (1 year nonresponse of treatment with UDCA); the odds ratio(OR) and 95% confidence interval(CI) were used to evaluate the risk.

## Results

### Baseline characteristics of the study population

During the study period, 257 PBC patients met the inclusion criteria and were included in the study (Table 1). The average patient age was 56.20 ± 11.84 years, including 223 (86.80%) female patients. A total of 235 (91.40%) PBC patients were subjected to the first UDCA treatment. A total of 83 (31.90%) had cirrhosis at baseline. Additionally, 132 (51.40%) patients were positive for serum antimitochondrial antibody (AMA) or the M2 subtype, and the patients were positive for other PBC-specific antibodies, including anti-sp100 25(9.70%) and anti-gp210 64(24.90%). Patients sought medical attention for fatigue in 18 cases (7.0%) and puritus in 36 cases (14.0%) at baseline.

**Table 1:** Baseline characteristics of the biochemical response in patients with PBC

Characteristics	Total (*n* = 257)	Non-responders (*n* = 87)	Responders (*n* = 170)	*P-*value
Sex(female)	223 (86.80)	73 (83.90)	150 (88.20)	0.273
Age (years)	56.20 ± 11.84	55.23 ± 10.97	56.74 ± 12.31	0.336
BMI	22.22 (20.44–24.09)	21.64 (19.56–24.23)	22.60 (20.70–24.23)	0.141
F-UDCA	235 (91.40)	80 (92)	155 (91.20)	0.822
Cirrhosis	83 (31.90)	33 (37.90)	49 (28.80)	0.147
Fatigue	18(7.0%)	8(9.20%)	10(5.90%)	0.439
Pruritus	36(14.0%)	13(14.90%)	23(13.50%)	0.850
Comorbidities				
Hypertension	29 (11.30)	5 (5.75)	24 (14.12)	0.043
Thyroid disease	50 (19.50)	11 (12.64)	39 (22.94)	0.046
Atherosis	5 (1.90)	4 (4.60)	1 (0.59)	0.028
Complications				
Esophageal and gastric varices	46 (17.90)	23 (26.44)	23 (13.53)	0.011
Ascites	39 (15.20)	20 (22.99)	19 (11.18)	0.038
Laboratory date				
Albumin (g/l)	36.90 (32.00–41.35)	33.80 (28.30–38.60)	38.20 (33.60–42.65)	< 0.001
Globulin (g/l)	35.13 ± 7.70	36.49 ± 7.80	34.45 ± 7.59	0.045
Total bilirubin (umol/l)	25.00 (15.5–49.30)	36.70 (24.40–70.10)	19.60 (13.60–34.35)	< 0.001
Alanine aminotransferase (×ULN)	60.00 (32.00–104.00)	75.00 (41.00–103.00)	51.00 (27.00–103.00)	0.019
Aspartate aminotransferase(×ULN)	69.00 (42.00–117.00)	88.00 (71.00–130.00)	54.00 (35.00–105.00)	0.010
Alkaline phosphatase (×ULN)	220.00 (132.00–347.50)	395.00 (259.00–549.00)	160.00 (113.00–233.50)	< 0.001
γ-Glutamyl transferase (×ULN)	206.00 (77.00–421.50)	400.00 (201.00–709.00)	159.00 (49.50–311.00)	< 0.001
Total cholesterol (mmol/l)	4.83 (3.90–6.06)	57.90 (24.10–170.60)	21.30 (8.35–68.80)	< 0.001
HDL-C (mmol/l)	1.28 (0.93–1.68)	1.29 (0.85–1.91)	1.26 (0.99–1.64)	0.676
LDL-C (mmol/l)	2.74 (2.09–3.64)	3.16 (2.32–4.72)	2.54 (1.95–3.33)	< 0.001
White blood cell (× 10^9^/l)	4.88 (3.59–5.97)	4.85 (3.82–6.39)	4.88 (3.58–5.74)	0.287
Neutrophil counts (×10^9^/l)	2.75 (1.92–3.65)	2.91 (2.12–4.28)	2.58 (1.89–3.44)	0.017
Lymphocyte counts (10^9^/l)	1.48 (1.04–1.92)	1.38 (1.03–1.78)	1.53 (1.06–2.05)	0.070
Monocyte counts (×10^9^/l)	0.31 (0.23–0.40)	0.31 (0.23–0.41)	0.31 (0.24–0.40)	0.885
NLR	1.83 (1.27–2.76)	2.16 (1.43–3.33)	1.70 (1.18–2.53)	0.001
Platelet count (×10^9^/l)	150.00 (93.50–225.00)	150.00 (85.00–228.00)	153.00 (95.50–222.00)	0.722
International normalized ratio	1.05 ()0.95–1.19	1.02 (0.92–1.18)	1.05 (0.97–1.20)	0.076
APTT(s)	35.80 (31.70–39.70)	37.29 ± 7.29	34.76 ± 6.10	0.004
Serum creatinine (umol/l)	58.00 (50.00–66.00)	54.00 (46.00–62.00)	60.00 (54.00–66.00)	< 0.001
Serum sodium (mmol/l)	139.80 (137.73–14,158)	139.10 (137.20–141.00)	140.30 (138.30–142.00)	0.008
Positive AMA-2	132 (51.40)	49 (56.32)	83 (48.82)	0.274
SP100	25(9.70)	9(10.34)	16(9.41)	0.823
GP210	64(24.90)	27(31.03)	37(21.76)	0.110

*BMI* body mass index, *NLR* neutrophil-to-lymphocyte ratio, *AMA* antimitochondrial antibody, *HDL-C* high-density lipoprotein cholesterol, *LDL-C* low-density lipoprotein cholesterol, *UDCA* ursodeoxycholic acid, *APTT* activated partial thromboplastin time, *ULN* upper limit of normal

We compared the clinical characteristics and parameters between the following groups: a response group (with ALP ≤ 1.67 times) and an inadequate response group (with ALP > 1.67 times the ULN). Overall, 170 (66.15%) patients did not response to UDCA treatment. BMI media (first quartile; third quartile) of the responder group 22.60 (20.70–24.23) were slightly higher than those of the non-responder group 21.64 (19.56–24.23) at baseline (BMI:). Furthermore, the differences among groups in gender, age, and liver cirrhosis were not statistically significant (*P* > 0.05). Interestingly, the complications of PBC, including ascites and esophageal varices, were significantly more frequent in the non-responder group than in the responder group (*P* < 0.05). A more significant increase in ALT, AST, ALP, GGT, total bilirubin (TB), and NLR levels after 12-months of UDCA treatment was noted in the inadequate response group than in the response group (*P* < 0.05). The definitive diagnosis of 73 patients was made by liver biopsy, with histological Scheuer classification stages as follows: I (*n* = 5), II (*n* = 24), III (*n* = 25), and IV (*n* = 19) (data not shown).

### NLR is an independent risk factor for non-response to UDCA treatment in PBC patients: binary logistic regression model results

Univariate binary logistic regression analysis showed that globulin, total bile acid, γ-glutamyl transferase (GGT), low-density lipoprotein (LDLC), total cholesterol (TC), neutrophils, APTT, and NLR were risk factors for UDCA nonresponse (*P* < 0.05). In the multivariate binary logistic regression analysis, we developed three models adjusted for different covariates to confirm the stability of the results. Model A, which was adjusted for age, sex, and BMI, indicated that NLR is an independent risk factor for the lack of response to UDCA treatment (OR: 1.355, 95% CI: 1.132–1.622, *P* = 0.001). Model B, which was adjusted for age, sex, BMI, hypertension, arterial plaque, and thyroid disease suggested that hypertension (OR: 3.704, 95% CI:1.137–12.062, *P* = 0.030) and NLR (OR: 1.402, 95% CI: 1.169–1.683, *P* < 0.001) were risk factors for the lack of response. Model C, which was adjusted for age, sex, BMI, hypertension, arterial plaque, thyroid disease, jaundice, albumin, globulin, total bile acid, total bilirubin, ALP, GGT, HDLC,LDLC, total cholesterol, triglycerides, and APTT, showed that ALB(OR:0.883,95% CI:0.806–0.967) level was the protective factors of the response, whereas the baseline ALP(OR:1.013, 95% CI:1.009–1.018), and NLR (OR:1.442, 95% CI:1.040–2.00, *P* = 0.013) levels were also risk factors for the lack of response to UDCA treatment (Table 2).

**Table 2:** Binary logistic regression analysis results of NLR and one-year lack of response to UDCA treatment in PBC patients

Variable	Univariate analysis		Multivariate analysis					
			Model A		Model B	Model C		
	OR (95% CI)	*P*-value	OR (95% CI)	*P*-value	OR (95% CI)	*P*-value	OR (95% CI)	*P*-value
Age (years)	0.989 (0.968–1.011)	0.335	0.988 (0.966–1.011)	0.317	0.993 (0.970–1.018)	0.598	1.011(0.969–1.055)	0.606
Sex (male/female)	0.660 (0.314–1.391)	0.275	1.609 (0.749–3.454)	0.223	1.119 (0.495–2.532)	0.787	1.191(0.327–4.348)	0.791
BMI	0.940 (0.864–1.022)	0.144	0.947 (0.868–1.032)	0.211	0.972 (0.891–1.060)	0.520	1.038(0.914–1.180)	0.565
Hypertension	2.714 (0.998–7.385)	0.051			3.704 (1.137–12.062)	0.030	1.860(0.403–8.595)	0.427
Arterial plaque	8.096 (0.891–7.585)	0.063			0.101 (0.010–1.022)	0.052	0.427(0.032–5.732)	0.521
Thyroid disease	0.482 (0.233–0.998)	0.049			2.122 (0.965–4.666)	0.061	20,639(0.869–8.013)	0.087
Jaundice	0.505 (0.258–0.988)	0.046					0.393(0.095–1.618)	0.196
Albumin (g/l)	0.903 (0.865–0.942)	0.000					0.883(0.806–0.967)	0.008
Globulin (g/l)	1.035 (1.000–1.071)	0.047					0.980(0.924–1.039)	0.489
Total bile acid (u/mol/L)	1.003 (1.001–1.005)	0.005					1.001(0.995–1.007)	0.759
Total bilirubin (umol/L)	1.003 (0.999–1.007)	0.129					0.986(0.972–1.001)	0.060
Alkaline phosphatase (u/l)	1.011 (1.008–1.014)	0.000					1.013(1.009–1.018)	< 0.001
γ-Glutamyl transferase (u/l)	1.003 (1.002–1.004)	0.000					0.999(0.997–1.001)	0.212
HDL-C (mmol/l)	1.313 (0.851–2.025)	0.218					1.346(0.403–4.488)	0.629
LDL-C (mmol/L)	1.325 (1.110–1.582)	0.002					0.531(0.232–1.215)	0.134
Total cholesterol (mmol/L)	1.278 (1.117–1.461)	0.000					1.722(0.866–3.423)	0.121
Triglycerides (mmol/L)	1.127 (0.879–1.446)	0.346					1.316(0.649–2.671)	0.447
Neutrophils (×10^9^/l)	1.359 (1.127–1.638)	0.001					1.012(0.691–1.481)	0.951
APTT(s)	1.066 (1.019–1.116)	0.006					1.054(0.977–1.137)	0.175
NLR	1.325 (1.109–1.584)	0.002	1.321 (1.104–1.580)	0.002	1.369 (1.141–1.643)	0.001	1.442(1.040–2.000)	0.028

Model A: adjusted for age, sex, and BMI

Model B: adjusted for age, sex, BMI, hypertension, arterial plaque, and thyroid disease

Model C: adjusted for age, sex, BMI, hypertension, arterial plaque, thyroid disease, jaundice, albumin, globulin, total bile acid, total bilirubin, ALP, GGT, HDLC, LDLC, total cholesterol, triglycerides, and APTT

## Discussion

In this retrospective study, we adopted multivariate analysis and adjusted confounding factors to identify the risk factors of UDCA nonresponse. We found that NLR was an independent risk factor for incomplete response to UDCA treatment in PBC patients. In addition, the NLR is advantageous due to its simple and rapid determination. To our knowledge, this report is the first to provide evidence that a high NLR level at baseline is associated with a future risk of poor response to UDCA treatment.

In our study, we adjusted for age, sex, BMI, hypertension, arterial plaque, thyroid disease, jaundice, albumin, globulin, total bile acid, total bilirubin, ALP, GGT, HDLC, LDLC, total cholesterol, triglycerides, and APTT. The baseline NLR (OR:1.442, 95% CI:1.040–2.00, *P* = 0.013) levels were risk factors for the lack of response to UDCA treatment. The NLR is a composite marker of absolute peripheral neutrophil and lymphocyte counts. Elevated NLR which implies higher inflammatory burden signifies high neutrophil count due to active inflammation and a low lymphocyte count correlates with defective response to the inflammatory process [19]. Neutrophil levels are associated with inflammation, whereas lymphocytes are indispensable for immunosurveillance and immunoediting [20]. Therefore, analysing them apart may miss the interactions between these subtypes. Multiple previous studies have suggested that the NLR is a value that has been recently identified in relation to systemmation, and has been extensively studied in cardiovascular, malignancy, and pancreatitis [21–23]. A high NLR that may be predictive for primary liver cancer invasion and metabasis indicates a poor prognosis for patients with primary liver cancer [24]. Meanwhile, NLR as an advanced histological or prognostic predictor in hepatitis B and C and nonalcoholic fatty liver disease has been reported previously [25–27]. More importantly, a cohort study established the value that a high NLR was associated with a poorer long-term prognosis (15 years) in the PBC patients [28]. The results of this study are consistent with our findings. Currently, there are still no studies that prove whether a high NLR is correlated with worse response to UDCA therapy.

It is worth mentioning that inflammation is one of the major pathophysiological mechanisms for the development and progression of PBC [29]. Immune cells, inflammatory cytokines, and chemokines have been found to be involved in the pathogenesis of PBC [30]. Neutrophils have been described as a major target of IL-8. In a previous study, high positive expression of IL-8 was observed in small bile ducts of PBC, especially in end-stage cirrhosis [31–33]. However, in the early stages of PBC and PSC, IL-8-positive bile ductules were relatively infrequent or completely absent [31]. Therefore, we speculate that neutrophilic infiltration could be related to the IL-8 expression in bile ductular epithelia. A previous study revealed that CD8+ and CD4+ lymphocytes were the predominant cell types among inflammatory cells within the portal area in PBC [34]. Compared to peripheral blood, liver tissue is a 100-fold enrichment of CD4 + T cells and a 10-fold enrichment of CD8 + T cells [35]. Previous studies have shown that UDCA responsders exhibited significantly less CD4 + T cell infiltration after UDCA treatment than before in PBC liver specimens [36]. Therefore, a decrease in the number of liver-infiltrating lymphocytes (CD4+ Th1 cells) is associated with a good response of patients to UDCA treatment. Besides, available data suggest that risk factors for nonresponse among patients of UDCA treatment include male sex, age at diagnosis, increased titers of IgG, AIH features, and vitamin D deficiency [37–39]. It is worth noting that some patients with PBC who do not respond to ursodeoxycholic acid therapy may be combined with autoimmune hepatitis. The overlap syndrome of PBC and AIH patients characterized with high IgG, ALT, or AST level and interface hepatitis, is also associated with the poor response and prognosis [38, 40]. It is well known that when patients are diagnosed with PBC-AIH overlap syndrome (OS), immunosuppressive therapy is necessary in addition to treatment with UDCA. Besides, some studies suggest that patients with a symptomatic presentation (fatigue and/or pruritus) may have a poorer response to UDCA therapy, and a worse prognosis than asymptomatic patients [41, 42]. Because fatigue and pruritus are extremely subjective symptoms, patients are often unable to report precisely in the everyday clinical practice, and records with unsatisfactory data were not excluded in our study. In clinical practice, if patients are intolerant to UDCA or do not have a sufficient biochemical response to treatment, a second-line therapy in the form of added obeticholic acid can be considered. Fibrates (fenofibrate and bezafibrate), which are not approved for PBC treatment are also used in some countries because of evidence of their beneficial effects [43]. Currently, there are no available biomarkers to identify the patients who are unlikely to respond to UDCA and who might benefit from early introduction of second-line therapy.

This retrospective cohort study established a relatively good predictor for the inadequate response of UDCA treatment. However, our current study has some inevitable limitations. First, this was a single-center study that needed more centers and more samples to further test. Second, because of the short-term follow-up duration, we also could not identify the predictive value for long-term outcomes. Third, the subjective feelings of fatigue and pruritus at the baseline were difficult to obtain precisely.

In conclusion, our findings suggest that the NLR is an independent risk factor for non-response to UDCA treatment in PBC patients.

## Data Availability

The data that support the findings of this study are available on request from the corresponding author. The data are not publicly available due to privacy or ethical restrictions.
